# Inhibition of ACE Retards Tau Hyperphosphorylation and Signs of Neuronal Degeneration in Aged Rats Subjected to Chronic Mild Stress

**DOI:** 10.1155/2015/917156

**Published:** 2015-11-30

**Authors:** Said AbdAlla, Ahmed el Hakim, Ahmed Abdelbaset, Yasser Elfaramawy, Ursula Quitterer

**Affiliations:** ^1^Molecular Pharmacology Unit, Department of Chemistry and Applied Biosciences, ETH Zurich, Winterthurerstrasse 190, 8057 Zurich, Switzerland; ^2^International Neuroscience Institute (INI) Hannover, Rudolf-Pichlmayr-Strasse 4, 30625 Hannover, Germany; ^3^Medical Research Centre (MRC), Ain Shams University Hospitals, Abassia, Cairo 11591, Egypt; ^4^Institute of Pharmacology and Toxicology, Department of Medicine, University of Zurich, Winterthurerstrasse 190, 8057 Zurich, Switzerland

## Abstract

With increasing life expectancy, Alzheimer's disease (AD) and other types of age-associated dementia are on the rise worldwide. Treatment approaches for dementia are insufficient and novel therapies are not readily available. In this context repurposing of established drugs appears attractive. A well-established class of cardiovascular drugs, which targets the angiotensin II system, is such a candidate, which currently undergoes a paradigm shift with regard to the potential benefit for treatment of neurodegenerative symptoms. In search for additional evidence, we subjected aged rats to chronic unpredictable mild stress, which is known to enhance the development of AD-related neuropathological features. We report here that four weeks of chronic mild stress induced a strong upregulation of the hippocampal angiotensin-converting enzyme (*Ace*) at gene expression and protein level. Concomitantly, tau protein hyperphosphorylation developed. Signs of neurodegeneration were detected by the significant downregulation of neuronal structure proteins such as microtubule-associated protein 2 (*Map2*) and synuclein-gamma (*Sncg*).* Ace* was involved in neurodegenerative symptoms because treatment with the brain-penetrating* ACE* inhibitor, captopril, retarded tau hyperphosphorylation and signs of neurodegeneration. Moreover,* ACE* inhibitor treatment could counteract glutamate neurotoxicity by preventing the downregulation of glutamate decarboxylase 2 (*Gad2*). Taken together,* ACE* inhibition targets neurodegeneration triggered by environmental stress.

## 1. Introduction

Alzheimer's disease (AD) is the most common form of dementia. While genetic factors are causally linked to familial AD, the pathogenesis of the predominant late-onset sporadic AD is diverse and less defined. Age is one of the best-documented risk factors for sporadic AD, which acts in concert with a wide array of brain insult-promoting vascular and metabolic factors such as hypertension, ischemia, diabetes, high cholesterol, and different forms of environmental stress and stress-related psychiatric symptoms, that is, depression and anxiety [1–3].

Experimental models of AD often reproduce genetic alterations whereas the impact of additional brain-damaging factors is more difficult to assess. The chronic unpredictable mild stress (CUMS) model imitates psychiatric risk factors such as psychological, psychosocial, and physical stress [4]. In agreement with the well-established impact of stress on neuronal viability, the model promotes signs of neuronal degeneration such as decreased synaptic transmission at hippocampal CA1-CA3 synapses, impaired neurogenesis, and cognitive dysfunction [5–7]. The sensitivity to stress in this model increases with age, a major factor of AD pathogenesis [8]. The relationship to AD is further supported by several studies, which show that the CUMS procedure worsens disease progression in genetic AD mouse models [9–11].

Moreover, the chronic mild stress protocol induces major neuropathological hallmarks of AD, that is, enhanced *β*-amyloid (A*β*) generation, tau hyperphosphorylation, and the appearance of various other AD markers [9, 11, 12]. In this respect, the chronic mild stress model could complement genetic AD models because the CUMS model solely relies on endogenously expressed proteins. In view of the recent failure of large clinical trials, which targeted *β*-amyloid plaques [13, 14], such alternative models, which imitate the disease process before the accumulation of A*β* plaques, could become of substantial value.

Inefficient A*β* plaque-targeting approaches also raise the necessity to identify players in AD pathogenesis, which could interrupt the disease process at an earlier stage [15]. For one player in AD pathogenesis a paradigm shift is currently on the way, which is the angiotensin system, notably the angiotensin-converting enzyme (*ACE*). While many studies considered* ACE* primarily as an enzyme that could promote proteolysis of *β*-amyloid in vitro [16–18], recent experimental and clinical studies provide strong evidence that inhibition of* ACE*-dependent angiotensin II generation in vivo could actually reduce signs of neurodegeneration in experimental AD models [19, 20] and slow the cognitive decline of patients with Alzheimer's disease [21–25].

In view of this emerging development, we aimed to further analyse the impact of* ACE* inhibition on early signs of neurodegeneration and applied the chronic unpredictable mild stress model in our study.

## 2. Materials and Methods

### 2.1. Chronic Unpredictable Mild Stress Model

Male Wistar rats (15 months of age) were subjected to the chronic unpredictable mild stress (CUMS) battery for four weeks. The CUMS protocol was performed essentially as described previously [8, 9]. After four weeks of stress, more than 90% of stressed rats showed signs of anhedonia as documented by a decrease in sucrose preference compared to nonstressed controls (i.e., <50% of sucrose consumption compared to nonstressed controls). The age-matched control group was housed under standard conditions and had free access to food and water. The captopril treatment group received captopril in drinking water (50 mg/kg/day, dissolved fresh every day) during the CUMS protocol. After 28 days of stress, at an age of 16 months, 4 h before the beginning of the dark phase, rats were anesthetized with ketamine and xylazine (100 mg/kg and 10 mg/kg, i.p.), and brains were removed and processed for immunohistology [9, 20]. For whole genome microarray gene expression profiling, biochemical analyses, and immunoblotting, the hippocampus was dissected out on ice and processed for further use as detailed below.

All animal experiments were performed in accordance with the NIH guidelines and were approved by the local committee on animal research (MRC, Ain Shams University Hospitals, Cairo, Egypt).

### 2.2. Whole Genome Microarray Gene Expression Profiling of Hippocampal Gene Expression

For whole genome microarray gene expression analysis, total hippocampal RNA was isolated from stressed rats and age-matched nonstressed control rats. Total RNA was processed for whole genome microarray gene expression profiling and hybridized to the GeneChip Rat Genome 230 2.0 Array (more than 31 000 probe sets, Affymetrix) as detailed previously [20]. The total RNA from 3 animals was pooled for one gene chip, and two gene chips are presented for each group. Microarrays were scanned with the Affymetrix GeneChip Scanner 7G, and signals were processed with a target value of 300 using GCOS (version 1.4, Affymetrix). Applied selection criteria for differently expressed genes (2-fold change requirement, just alpha, no false discovery correction, and *P* < 0.05) were validated specifically for drug treatment effects [20] and follow the guidelines of the Microarray Quality Control (MAQC) project for the identification of reproducible gene lists [26, 27]. Probe sets with significant difference (*P* < 0.05 and ≥2-fold difference, with call present and/or signal intensity ≥100) between stressed rats and nonstressed, age-matched control rats were used for GO classification. Microarray data are available at the NCBI GEO repository (GSE72062).

Gene expression of* Ace* and* Map2* in the hippocampus of stressed rats and nonstressed controls was determined by real-time quantitative (q) RT-PCR with a LightCycler 480 (Roche Diagnostics). Sequences of the forward and reverse primers were as follows:* Ace* forward 5′-GATTGCAGCCGGGCAACTTTTC-3′;* Ace* reverse 5′-CGGATCCGATGATCCTTCGC-3′;* Map2* forward 5′-CACTGGAAGAAGCCTCGAAGA-3′;* Map2* reverse 5′-CACGGGCATTTCGATGAACC-3′.

### 2.3. Immunoblotting, Immunohistology, and Biochemical Methods

Immunoblot detection of hippocampal proteins was performed with hippocampal tissue extracts as described [9, 20]. For immunohistology and immunofluorescence analysis, we used paraffin-embedded brain sections obtained from stressed and nonstressed rats (8 *μ*m, taken at 50 *μ*m intervals, 10–15 sections per set). The following antibodies were used for immunoblotting, immunofluorescence, and immunohistology: anti-Ace antibodies raised in rabbit against an antigen corresponding to amino acids 720–750 of mouse Ace [20]; anti-angiotensin II antibodies raised in rabbit against synthetic angiotensin II [20]; anti-APP/A*β* antibodies raised in rabbit against a peptide corresponding to amino acids 672–714 of human APP [20]; anti-AT1R antibodies raised in rabbit against an antigen corresponding to amino acids 306–359 of the mouse Agtr1a sequence [20]; anti-Gad65 antibody (clone N-GAD65, developed in mouse against a synthetic peptide corresponding to amino acids 4–22 of human GAD65); anti-Grin3a antibodies raised in rabbit against a synthetic peptide derived from the extracellular domain of rat Grin3a (Abcam); anti-MAP2 mouse monoclonal antibody generated against bovine microtubule-associated protein, MAP2 (Clone AP-20); anti-chondroitin sulfate proteoglycan NG2 mouse monoclonal antibody generated against a truncated form of NG2 (MAB5384); anti-PHF-tau pSer202 and pThr 205 antibody (mouse monoclonal antibody, clone AT8; Pierce); and anti-Sncg antibodies raised in goat against an internal region of human SNCG (Santa Cruz Biotechnology).

Hippocampal* Ace* activity was assessed with a fluorogenic substrate (Abz-FRK(Dnp)-P; Biomol) as described [20], and determination of hippocampal glutamic acid decarboxylase (Gad) enzyme activity was performed as detailed previously [8].

### 2.4. Statistical Analysis

The results are presented as means ± s.d. We used unpaired two-tailed Student's *t*-test for comparisons between two groups. Statistical significance was set at a *P* value of <0.05, unless otherwise specified.

## 3. Results

### 3.1. Chronic Unpredictable Mild Stress Induced Upregulation of Hippocampal Ace Protein and Gene Expression

**Figure 1:**
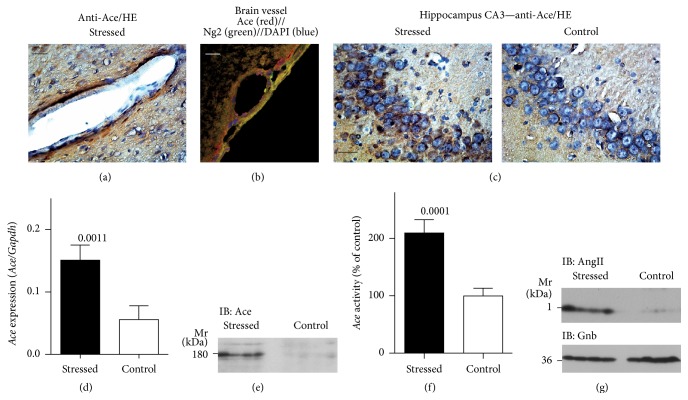
Chronic unpredictable mild stress induced upregulation of hippocampal Ace protein and gene expression. (a) Immunohistological localization of Ace in a brain vessel of a stressed rat. Nuclei were stained with hematoxylin (HE); bar: 20 *μ*m. (b) Immunofluorescence localization of Ace (red) and the pericyte marker Ng2 (green) in a brain vessel of a stressed rat. Ace was detected with affinity-purified rabbit anti-Ace antibodies followed by F(ab)_2_ fragments of Alexa Fluor 546-labeled (red) secondary antibodies, and Ng2 was detected with affinity-purified murine anti-NG2 antibody followed by F(ab)_2_ fragments of Alexa Fluor 488-labeled (green) secondary antibodies. Nuclei were stained with DAPI (bar: 20 *μ*m). (c) Immunohistological localization of Ace in hippocampal CA3 neurons of a stressed rat (left) relative to a nonstressed control (right). Nuclei were stained with hematoxylin (HE); bar: 20 *μ*m. Histological experiments are representative of 4 rats/group (a–c). (d) Hippocampal* Ace* gene expression was determined by qRT-PCR and is presented as the ratio of* Ace*/*Gapdh* expression (±s.d.; *n* = 4; *P* = 0.0011). (e) Immunoblot detection of the hippocampal Ace protein with anti-Ace antibodies in stressed rats relative to nonstressed controls (*n* = 4/group). (f) Hippocampal* Ace* activity of stressed rats relative to nonstressed controls (i.e., 100%; ±s.d., *n* = 8; *P* = 0.0001). (g) The hippocampal angiotensin II content was determined by immunoblot in stressed rats relative to nonstressed controls (upper panel). The lower panel shows a control immunoblot, which detects Gnb (*n* = 4/group).

Chronic stress worsens the progression of AD in patients and transgenic disease models [9–11] but underlying mechanisms are incompletely understood. Because the angiotensin-converting enzyme (*ACE*) is upregulated in brains of AD patients and mice [20], we asked for an interrelationship between* Ace* and stress. We used the chronic unpredictable mild stress model to investigate the role of* Ace* in stress-induced signs of neurodegeneration. Initially, we analysed the localization of* Ace* in stressed and nonstressed rat brain. Complementary to previous data with AD mice [20], endothelial cells lining a brain vessel of a stressed rat showed strong immunostaining for* Ace* (Figure 1(a)). Immunofluorescence localization of the pericyte marker, chondroitin sulfate proteoglycan* Ng2*, indicated that* Ace* was predominantly present in vascular endothelial cells whereas adjacent pericytes showed weaker staining for* Ace* (Figure 1(b)).

We further investigated the localization of* Ace* by immunohistology and focused on the hippocampal area because chronic mild stress induces profound changes at hippocampal CA3-CA1 synapses [7], that is, the brain area, which is highly susceptible to AD-related neuronal damage. In agreement with previous results [20], immunohistology analysis detected Ace immunoreactivity in cell bodies of hippocampal CA3 neurons (Figure 1(c)). Moreover, immunohistology revealed a strong increase in Ace immunoreactivity in hippocampal CA3 neurons of a stressed rat relative to that of a nonstressed control (Figure 1(c)).

In agreement with the increased Ace immunoreactivity, the gene expression level of hippocampal* Ace* was also significantly increased in stressed rats, that is, the hippocampal* Ace* gene expression level was 2.72 ± 0.43-fold higher in stressed rats compared to that in nonstressed controls (*P* = 0.0011; Figure 1(d)). Concomitantly, with* Ace* gene expression, the increased hippocampal Ace protein level of stressed rats was detected in immunoblot (Figure 1(e)). The increase in Ace protein was accompanied by a significantly elevated hippocampal* Ace* activity; that is, the hippocampal* Ace* activity was 2.09 ± 0.24-fold higher (*P* = 0.0001) in stressed rats compared to that in nonstressed controls (Figure 1(f)). The elevated* Ace* activity was also reflected by an increased hippocampal angiotensin II content in stressed rats relative to nonstressed controls (Figure 1(g)). Taken together, chronic unpredictable mild stress led to a significant upregulation of the Ace protein in the hippocampal area of stressed rats. As a consequence of* Ace* upregulation, the hippocampal angiotensin II content in stressed rats was elevated compared to that in nonstressed controls.

### 3.2. Tau Hyperphosphorylation in the Hippocampus of Stressed Rats

**Figure 2:**
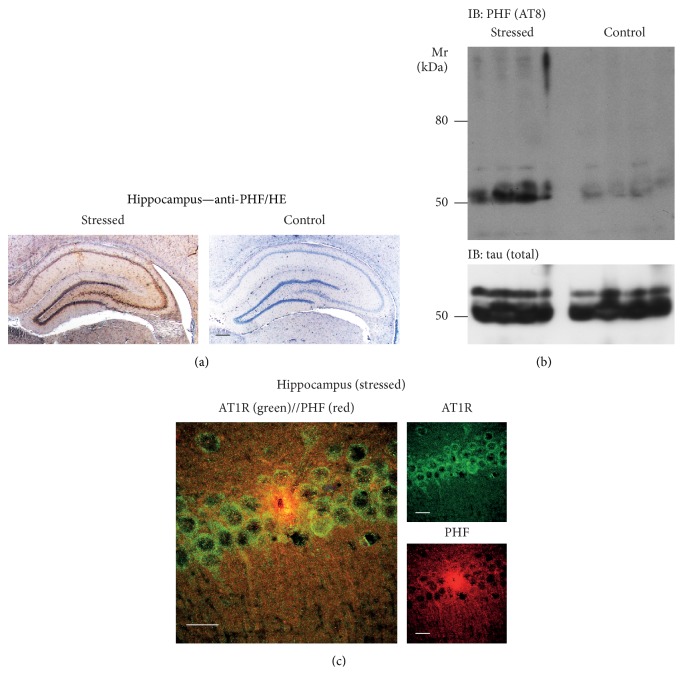
Tau hyperphosphorylation in the hippocampus of stressed rats. (a) Immunohistological detection of the hyperphosphorylated tau protein (anti-PHF) in the hippocampus of a stressed rat (left panel) relative to a nonstressed control (right panel). Nuclei were counterstained with hematoxylin (HE; bar: 200 *μ*m). (b) Immunoblot detection of hyperphosphorylated tau in hippocampal extracts of stressed rats relative to nonstressed controls was performed with anti-PHF antibody (IB: PHF (AT8)). The lower panel is a control immunoblot detecting total tau protein (*n* = 4/group). (c) Immunofluorescence localization of AT1R (green) and PHF (red) in hippocampal CA1 neurons of a stressed rat (bar: 20 *μ*m). The AT1R was detected with affinity-purified rabbit anti-AT1R antibodies followed by F(ab)_2_ fragments of Alexa Fluor 488-labeled (green) secondary antibodies, and hyperphosphorylated tau was visualized with affinity-purified mouse anti-PHF antibody followed by F(ab)_2_ fragments of Alexa Fluor 546-labeled secondary antibodies (red). Histological experiments are representative of 4 rats/group ((a) and (c)).

The strong upregulation of* Ace* in the hippocampus of stressed rats prompted us to search for signs of hippocampal neurodegeneration because previous data showed a causal relationship between* Ace* upregulation and signs of neurodegeneration in a genetic model of AD [20]. In this context we determined the level of tau hyperphosphorylation as a hallmark of AD. Immunohistological analysis demonstrated that four weeks of chronic unpredictable mild stress induced a strong increase in the level of phosphorylated tau protein in the hippocampus of an aged rat, as detected in a hippocampal section with anti-PHF (AT8) antibody (Figure 2(a)). Immunohistology also revealed that the increased tau phosphorylation was predominant in neurons of the CA1-CA3 region (Figure 2(a)). In contrast, PHF antibody staining was negligible in hippocampal neurons of the nonstressed control (Figure 2(a)).

Immunoblot detection of phosphorylated tau in hippocampal tissue extracts confirmed the immunohistology data and showed an increased level of hyperphosphorylated tau protein in stressed rats compared to that in nonstressed controls (Figure 2(b)). As an additional control, the total level of hippocampal tau protein was not different between stressed and nonstressed rats (Figure 2(b), lower panel). Taken together, the upregulation of* Ace* and angiotensin II in hippocampal neurons of stressed rats was accompanied by tau hyperphosphorylation.

*Ace*-dependent angiotensin II generation promotes signs of hippocampal neurodegeneration by activation of the AT1 receptor (AT1R) in a genetic AD model [20]. Complementary to those data, immunofluorescence analysis of a stressed rat brain showed AT1R immunoreactivity in hippocampal CA1 neurons, which also displayed accumulation of hyperphosphorylated tau protein (Figure 2(c)). Concomitantly, neuronal loss of a highly PHF-positive CA1 neuron was detected (Figure 2(c)). Thus, stress promoted* Ace*-dependent release of the AT1R-stimulating angiotensin II peptide together with tau hyperphosphorylation in hippocampal AT1R-positive neurons.

### 3.3. Whole Genome Microarray Gene Expression Profiling Detected Stress-Induced Signs of Hippocampal Neurodegeneration

**Figure 3:**
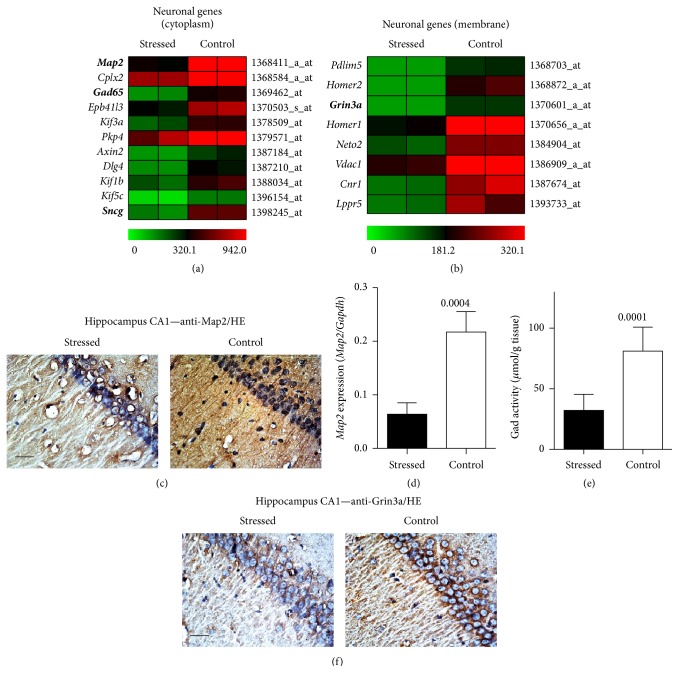
Whole genome microarray gene expression profiling detected stress-induced signs of hippocampal neurodegeneration. (a) and (b) Whole genome microarray gene expression profiling of hippocampal gene expression documents that stress promoted the significant downregulation of probe sets detecting neuron-specific genes with cytosolic localization (a) or membrane localization (b) according to GO analysis. All probe sets were significantly downregulated in the hippocampus of stressed rats relative to nonstressed controls (*P* < 0.05 and ≥2-fold difference). The heat map visualizes signal intensities (centered to the median value). (c) Immunohistological detection of Map2 in hippocampal CA1 neurons of a stressed rat (left panel) relative to a nonstressed control (right panel; bar: 20 *μ*m). (d) Hippocampal* Map2* expression level was determined by qRT-PCR and is presented as the ratio of* Map2*/*Gapdh* expression (±s.d.; *n* = 4; *P* = 0.0004). (e) Hippocampal Gad activity of stressed rats relative to nonstressed controls (±s.d.; *n* = 8; *P* = 0.0001). (f) The decreased hippocampal Grin3a protein level of a stressed rat (left panel) relative to that of a nonstressed control (right panel) was detected by immunohistology in hippocampal CA1 neurons; bar: 20 *μ*m. Immunohistology data are representative of 4 rats/group ((c) and (f)).

We further investigated the impact of stress on signs of neurodegeneration and performed whole genome microarray gene expression profiling of hippocampal gene expression. Gene ontology (GO) analysis searched for differently expressed neuron-specific genes by applying neuron-specific GO terms, that is, neuron, axon, dendrite, and synapse. Following established selection criteria for significantly different probe sets (≥2-fold difference and *P* < 0.05), the GO analysis documented that stress triggered the significant downregulation of neuron-specific genes, which were categorized according to the cellular localization of the respective proteins, that is, cytoplasm and membrane (Figures 3(a) and 3(b)).

The list of significantly downregulated genes encompasses major neuronal structure proteins such as microtubule-associated protein 2 (*Map2*) and synuclein-gamma,* Sncg* (Figure 3(a)). Because the loss of neuronal* Map2* is a characteristic feature of AD-related neurodegeneration, which is causally linked to stress-induced tau hyperphosphorylation [9, 28], we performed immunolocalization of the hippocampal* Map2*. Immunohistology analysis detected the decreased* Map2* content in hippocampal CA1 neurons of a stressed rat compared to that of a nonstressed control (Figure 3(c)). The stress-induced downregulation of the hippocampal Map2 protein was further confirmed by qRT-PCR (Figure 3(d)). Together these data indicate that chronic unpredictable mild stress led to tau hyperphosphorylation and decreased the level of neuron-specific genes such as* Map2*. The decreased expression of neuronal structure proteins complements previous data, which show that chronic mild stress triggers alterations of hippocampal synapses and promotes a decrease in dendritic spine density [7].

The expression of glutamic acid decarboxylase 2 (*Gad65/Gad2*) was also downregulated by chronic unpredictable mild stress (Figure 3(a)). The decreased* Gad65* expression could account for the significantly decreased hippocampal Gad enzyme activity, which was measured in hippocampal tissue extracts of stressed rats compared to that of nonstressed controls (Figure 3(e)). The ensuing decreased metabolism of the excitatory neurotransmitter, glutamate, could be partially responsible for the increased tau phosphorylation under chronic mild stress [8, 29]. In addition to the decreased glutamate degradation, the expression of the inhibitory and neuroprotective NMDA receptor subtype* Grin3a* (NR3A) [30, 31] was also downregulated by stress (Figure 3(b)). Immunohistology data complemented the microarray study and showed abundant membrane-localized Grin3a protein in hippocampal CA1 neurons of a nonstressed control whereas the neuronal Grin3a protein was barely detectable in the hippocampal neurons of a stressed rat (Figure 3(f)). As a consequence of stress-induced* Grin3a* (NR3A) downregulation, glutamate excitotoxicity, for example, triggered by decreased Gad enzyme activity, could be further augmented [30, 31]. Taken together, whole genome microarray gene expression profiling detected signs of neurodegeneration and markers of enhanced glutamate excitotoxicity in the hippocampus of stressed rats.

### 3.4. Treatment with the Brain-Penetrating* ACE* Inhibitor, Captopril, Retarded Signs of Neurodegeneration Induced by Chronic Mild Stress

**Figure 4:**
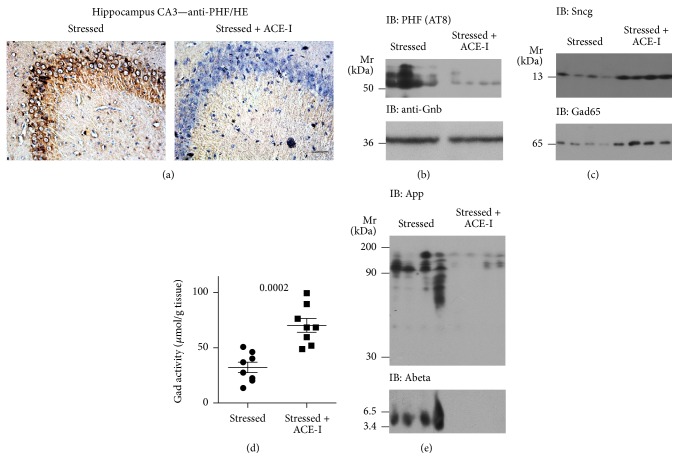
Treatment with the brain-penetrating* ACE* inhibitor captopril retarded signs of neurodegeneration induced by chronic mild stress. (a) Immunohistological detection of hyperphosphorylated tau protein with anti-PHF antibody in hippocampal CA3 neurons of a stressed rat without treatment (left panel) relative to a stressed rat treated with the* ACE* inhibitor captopril (Stressed + ACE-I) during the stress protocol (right panel); bar: 40 *μ*m. Immunohistological experiments are representative of 4 rats/group. (b) Immunoblot detection of hyperphosphorylated tau protein (IB: PHF (AT8)) in hippocampal extracts of stressed rats without treatment relative to stressed rats treated with the* ACE* inhibitor captopril (Stressed + ACE-I) during the stress protocol (*n* = 4/group). (c) Immunoblot detection of Sncg (upper panel) and Gad65 (lower panel) in hippocampal extracts of stressed rats without treatment relative to stressed rats with* ACE* inhibitor (Stressed + ACE-I) treatment (*n* = 4/group). (d) Hippocampal Gad activity of stressed rats without treatment relative to stressed rats treated with the* ACE* inhibitor (Stressed + ACE-I) captopril (*n* = 8/group; *P* = 0.0002). (e) Immunoblot detection of the amyloid beta (A4) precursor protein, App (IB: App; upper panel), and *β*-amyloid (IB: Abeta; lower panel) in hippocampal extracts of stressed rats without treatment relative to stressed rats treated with the* ACE* inhibitor captopril (Stressed + ACE-I) during the stress protocol (*n* = 4/group).

In view of the strongly upregulated Ace protein, we asked whether treatment with the brain-penetrating* ACE* inhibitor, captopril, could affect the process of neurodegeneration triggered by stress. Aged rats were treated with captopril during the chronic unpredictable mild stress procedure. As a sign of neurodegeneration, we determined the hippocampal tau phosphorylation. Immunohistology analysis revealed a substantially decreased level of hyperphosphorylated tau protein in hippocampal CA3 neurons of a stressed rat with captopril treatment relative to that of a stressed control without* ACE* inhibitor treatment (Figure 4(a)). Immunoblot detection of hyperphosphorylated tau confirmed immunohistology data and demonstrated the decreased hyperphosphorylated tau protein content in the hippocampus of stressed rats with captopril treatment relative to that of stressed rats without treatment (Figure 4(b)). Concomitantly, with the decreased tau phosphorylation, the stress-induced loss of synuclein-gamma (*Sncg*) was also retarded by* ACE* inhibition with captopril (Figure 4(c), upper panel).

We next assessed the hippocampal Gad65 protein level and activity. Immunoblot analysis showed that captopril treatment counteracted the stress-induced decrease in hippocampal Gad65 protein (Figure 4(c), lower panel). The preserved Gad65 protein was accompanied by a significantly higher hippocampal Gad activity in stressed rats with captopril treatment compared to that in stressed rats without treatment (Figure 4(d)). Taken together, our data indicate that inhibition of* ACE* with a brain-permeable* ACE* inhibitor retarded the stress-induced tau hyperphosphorylation and counteracted the downregulation of Gad activity triggered by chronic mild stress.

Previous studies indicate that chronic mild stress enhances the generation of A*β* as a major neuropathological feature of AD [12] while* ACE* inhibition decreases the hippocampal A*β* level, as documented in a genetic AD model [20]. The increased A*β* generation could be a consequence of the stress-induced increase in the steady-state level of the amyloid beta (A4) precursor protein (App) [32]. Complementary to those data, immunoblot analysis detected the high hippocampal App load in stressed rats without treatment whereas the hippocampal App content was substantially decreased in stressed rats with* ACE* inhibitor treatment (Figure 4(e), upper panel). Concomitantly, immunoblot analysis revealed that the hippocampal A*β* level was also decreased in stressed rats with ACE inhibitor treatment compared to that in stressed rats without treatment (Figure 4(e), lower panel).

## 4. Discussion

### 4.1. Chronic Unpredictable Mild Stress Triggered Signs of Hippocampal Neurodegeneration

Our study applied the chronic unpredictable mild stress model, which imitates major features of sporadic AD such as tau hyperphosphorylation, enhanced A*β* generation, and other signs of dendritic and synaptic degeneration [7, 12]. In agreement with those previous studies, we also found that chronic mild stress triggered signs of hippocampal neurodegeneration, which was documented by hippocampal tau hyperphosphorylation, decreased levels of major neuronal proteins such as* Map2* and* Sncg*, and an increased level of A*β*-generating App. Concomitantly, with AD-related signs of neurodegeneration, glutamate excitotoxicity-enhancing features appeared such as decreased Gad65 protein level and enzyme activity and decreased hippocampal expression of the glutamate-inhibitory NMDA receptor subtype,* Grin3a* (NR3A). The ensuing sensitization of glutamate excitotoxicity could contribute to stress-induced signs of neurodegeneration because glutamate excitotoxicity is a major factor, which contributes to neurodegeneration and tau hyperphosphorylation [29].

### 4.2. Chronic Mild Stress Induced Upregulation of Hippocampal Ace Protein and Activity

In search for additional stimuli, which could account for the development of stress-induced signs of neurodegeneration, our study detected a significantly increased hippocampal protein level of the angiotensin II-generating enzyme,* Ace*. Immunohistology analysis localized the upregulated Ace protein not only in brain vessels but also in neuronal cell bodies of the hippocampus. Concomitantly, an increased hippocampal* Ace* activity and angiotensin II peptide level were detected. The upregulation of neuronal* Ace* expression and protein level could be a direct consequence of the stress-induced activation of the hypothalamic pituitary adrenocortical (HPA) axis because several studies found that* ACE* expression was triggered by glucocorticoids and glucocorticoid receptor activation [33–35].

### 4.3. *ACE* Inhibition Retarded Signs of Neurodegeneration Induced by Chronic Mild Stress

The upregulated Ace protein could be causally involved in signs of neurodegeneration triggered by chronic mild stress because treatment with the brain-penetrating* ACE* inhibitor, captopril, retarded the development of signs of stress-induced neurodegeneration, that is, tau hyperphosphorylation, downregulation of neuronal proteins, decreased Gad enzyme activity, and increased hippocampal App level.

The mechanism underlying the neuroprotective activity of central* ACE* inhibition is not entirely understood. Several studies indicated that the neuroprotective effect of central* ACE* inhibition is largely due to a decrease in angiotensin II generation and subsequently blunted activation of the angiotensin II AT1 receptor, AT1R [20, 36–40]. Such conclusion is supported by data, which show the involvement of angiotensin II-AT1R in major neuropathological features such as tau hyperphosphorylation [37], enhanced glutamate release [38], and increased A*β* formation [20, 39, 40]. In addition to direct neuroprotection, inhibition of the* ACE*-AT1R axis could also counteract the stress response by blunting the release of ACTH [41]. In agreement with a role of the* ACE*-angiotensin II-AT1R axis in stress-induced neurodegeneration, we found that the hippocampal angiotensin II content was increased by stress, and hippocampal neurons with hyperphosphorylated tau protein and overt neurodegeneration were AT1R-positive.

In view of the stress-induced upregulation of the hippocampal* Ace* and the neuroprotective activity of* ACE* inhibition in the chronic mild stress model, our data support a causative role of* ACE* in stress-induced neurodegeneration. Because the model reproduces major features of sporadic AD (i.e., tau hyperphosphorylation, A*β* generation, and glutamate excitotoxicity), our study could provide a rationale for the documented efficacy of centrally active* ACE* inhibitors in several clinical studies, which showed retardation of AD-related cognitive decline as a major sign of neurodegeneration [21–25]. In this context, the suggested repurposing of* ACE* inhibitors for clinical conditions related to AD and other types of dementia [42] could be a potential option, which deserves further study.

## 5. Conclusion

Inhibition of* ACE* with the centrally acting captopril retarded the development of hippocampal tau phosphorylation, signs of neurodegeneration, and amyloid beta (A4) precursor protein (App) upregulation in rats subjected to chronic unpredictable mild stress, as a model, which reproduces major pathological features of sporadic AD.
